# KCTD20, a relative of BTBD10, is a positive regulator of Akt

**DOI:** 10.1186/1471-2091-14-27

**Published:** 2013-10-24

**Authors:** Mikiro Nawa, Masaaki Matsuoka

**Affiliations:** 1 Department of Pharmacology, Tokyo Medical University, 6-1-1 Shinjuku, Shinjuku-ku, Tokyo 160-8042, Japan

**Keywords:** BTBD10, Akt, KCTD20

## Abstract

**Background:**

BTBD10 binds to Akt and protein phosphatase 2A (PP2A) and inhibits the PP2A-mediated dephosphorylation of Akt, thereby keeping Akt activated. Previous studies have suggested that BTBD10 plays an important role in preventing motor neuronal death and accelerating the growth of pancreatic beta cells. Because levels of BTBD10 expression are much lower in many non-nervous tissues than nervous tissues, there may be a relative of BTBD10 that has BTBD10-like function in non-neuronal cells.

**Results:**

A 419-amino-acid BTBD10-like protein, named KCTD20 (potassium channel tetramerization protein domain containing 20), was to found to bind to all Akt isoforms and PP2A. Overexpression of KCTD20 increased Akt phosphorylation at Thr308, as BTBD10 did, which suggests that KCTD20 as well as BTBD10 positively regulates the function of Akt. KCTD20 was ubiquitously expressed in non-nervous as well as nervous tissues.

**Conclusions:**

KCTD20 is a positive regulator of Akt and may play an important role in regulating the death and growth of some non-nervous and nervous cells.

## Background

Akt (three isoforms named Akt1-3 in mammalian cells) plays an important role in promoting the survival of many cells. Phosphatidylinositol 3-kinase is activated in the growth factor-mediated signaling cascade, generating the secondary messengers phosphatidylinositol-3, 4-bisphosphate and phosphatidylinositol-3,4,5-trisphosphate (PIP_3_), which recruit Akt to the inner leaflet of the cytoplasmic membrane. Akt, anchored to the membrane via PIP_3_, is phosphorylated and activated by both 3-phosphoinositide-dependent kinase-1 (PDK-1)-mediated phosphorylation of Akt at Thr308 and PDK-2 (or Ser473 kinase)-mediated phosphorylation of Akt at Ser473. Activated Akt subsequently phosphorylates and activates downstream target proteins, thereby promoting cell survival [1,2].

An insufficiency of the Akt signaling has been assumed to contribute to the pathogenesis of various human diseases, including neurodegenerative diseases, stroke, cancer, and diabetes [1-6]. In amyotrophic lateral sclerosis (ALS), a representative motor-neuron-specific neurodegenerative disease, levels of Akt phospholylation have been reported to be diminished, which may lead to motor neuronal death [7,8].

BTBD10 is a unique Akt activator [9]. It activates Akt by binding to both Akt and PP2A and by inhibiting PP2A-mediated dephosphorylation of Akt. Overexpression of BTBD10 increases Akt phosphorylation, whereas loss-of-function of BTBD10 decreases Akt phosphorylation in neuronal and pancreatic beta cells. Consequently, overexpression of BTBD10 inhibits neuronal death caused by expression of a familial ALS-linked gene G93A-superoxide dismutase 1 (SOD1) [9]. Another study has shown that overexpression of BTBD10 (also named glucose metabolism-related protein 1) promotes the growth of pancreatic beta cells, whereas knockdown of endogenous BTBD10 expresion decreases high glucose-induced cell proliferation and insulin-stimulated Akt phosphorylation [10]. The level of BTBD10 expression is reduced in motor neurons in spinal cords of sporadic ALS patients [11] where TDP-43 aggregates are formed [12]. Disruption of the *btbd-10* gene has been shown to cause loss of motor neurons and impairment of motor performance in *Caenorhabditis elegans*[11]. These results suggest that reduction of BTBD10 expression may contribute to motor neuronal death. In addition, the level of BTBD10 expression has been shown to be downregulated in a rat intracerebral hemorrhage model [13].

Because the levels of BTBD10 expression are much lower in many non-nervous tissues than nervous tissues [9], there may be a relative of BTBD10 that has BTBD10 function in non-neuronal cells.

In the current study, we investigated KCTD20 (potassium channel tetramerization protein domain containing 20), an isoform of BTBD10 [14]. Similar to BTBD10, KCTD20 was found to associate with all Akt isoforms and PP2A and upregulate its phospholylation level at Thr308.

## Results

### KCTD20 is a relative of BTBD10

**Figure 1:**
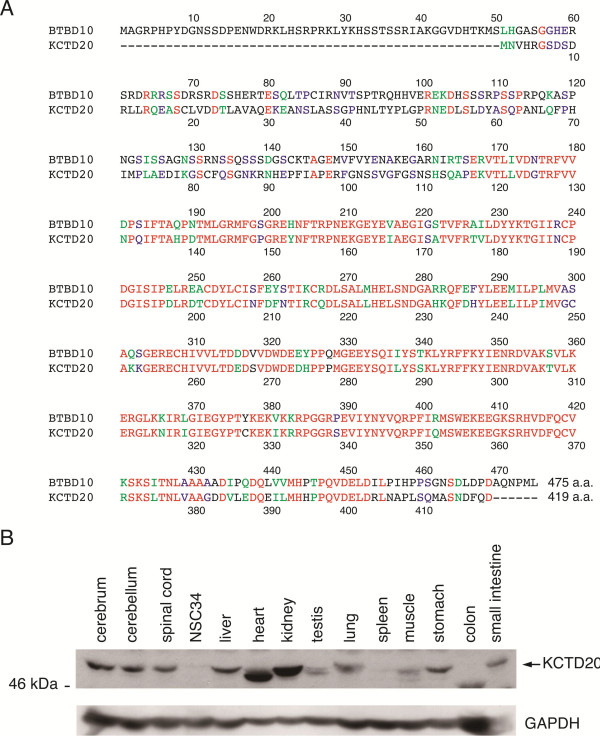
**Comparison in the amino acid sequence between BTBD10 and KCTD20 and KCTD20 expression in mouse tissues. A**, Amino acid sequences of human BTBD10 and human KCTD20 were aligned by ClustalW. Identical residues are shown by red characters while residues that are strongly similar or weakly similar are shown by green or blue characters, respectively. A dash (-) means the gap. **B**, Mouse tissue lysates were subjected to SDS-PAGE, followed by immunoblot analysis using anti-KCTD20 antibody (upper panel) or anti-GAPDH antibody (lower panel).

The gene encoding 419-amino-acid human KCTD20 is located in chromosome 6, while that encoding 475-amino-acid human BTBD10 is in chromosome 11. The overall similarity in the amino acid sequence between human BTBD10 and KCTD20 is 81.4% (Figure 1A). The C-terminal 330-amino-acid region of BTBD10 is responsible for the binding of BTBD10 to Akt [9]. The similarity in the amino acid sequence between the C-terminal 330-amino-acid regions of BTBD10 and KCTD20 is 91.4% (296/324) (Figure 1A). The *KCTD20* gene is highly conserved among different mammalian species. The similarity in the amino acid sequence between human [GenBank:NP_775833] and mouse [GenBank:NP_080164] KCTD20 is 94% (amino acids were aligned using BLAST2).

KCTD20 is ubiquitously expressed in mouse tissues, including nervous tissues (Figure 1B). Compared with BTBD10, levels of KCTD20 expression in non-nervous tissues except testis, spleen, and colon, are equal to or higher than those in nervous tissues.

### KCTD20 interacts with Akt or a catalytic subunit of PP2A

**Figure 2:**
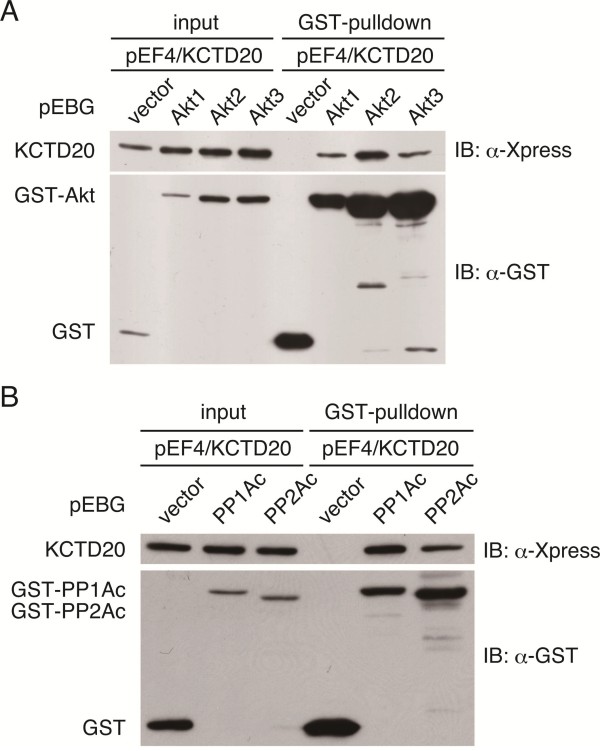
**Interaction between KCTD20 and Akt or a catalytic subunit of protein phosphatase in COS7 cells. A** and **B**, GST-Akt **(A)** or GST-protein phosphatase catalytic subunit **(B)** and His-Xpress-KCTD20 were coexpressed in COS7 cells by transfection. pEF4/KCTD20 encoded N-terminally His-Xpress-tagged KCTD20. pEBG vectors encoded N-terminally GST-tagged proteins (Akt1, Akt2, Akt3, the catalytic subunit of PP1A (PP1Ac), and the catalytic subunit of PP2A (PP2Ac)). The pEBG backbone vector (named vector) encoded GST. GST-pulldown assays with glutathione sepharose beads were performed using the cell lysates. His-Xpress-KCTD20, co-precipitated with GST-Akt **(A)** or GST-catalytic subunit of protein phosphatase **(B)**, was detected with anti-Xpress antibody (upper panel) and anti-GST antibody (lower panel).

BTBD10 binds to all Akt isoforms and upregulates their phosphorylation by inhibiting their dephosphorylation by PP2A [9]. GST-pulldown assays showed that KCTD20 was co-precipitated with GST-tagged Akt 1, 2 or 3 but not with GST (Figure 2). KCTD20 also co-precipitated with the GST-tagged catalytic subunit of PP1A and PP2A [9]. Theses results show that KCTD20 binds to all Akt isoforms, PP1A, and PP2A.

### Overexpression of KCTD20 upregulates the level of Akt phospholylation at Thr308

**Figure 3:**
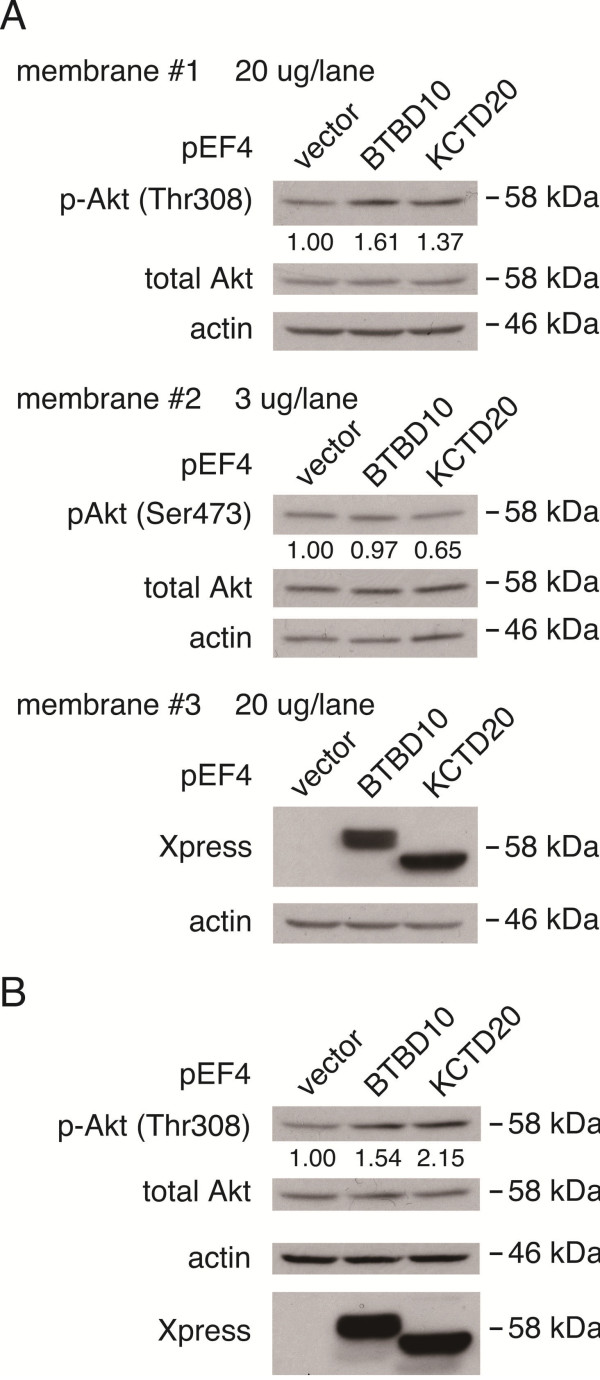
**KCTD20 upregulates the level of phospho-Akt in NSC34 cells. A**, NSC34 cells, transfected with pEF4-BTBD10, pEF4-KCTD20, or backbone vector, were harvested at 48 hr after transfection. The cell lysates were subjected to SDS-PAGE, followed by immunoblot analysis with indicated antibodies. **B**, The same experiment, shown in **(A)**, was repeated.

Based on the finding that KCTD20 interacts with all Akt isoforms and catalytic subunits of protein phosphatases, we next examined the effect of overexpression of KCTD20 on the level of Akt phosphorylation. NSC34 motor neuronal cells were transfected with an expression vector encoding BTBD10 or KCTD20. The level of Akt phosphorylation at Thr308 was increased by overexpression of BTBD10 as well as KCTD20 (Figure 3A) and this result was reproduced in another identical experiment (Figure 3B). In contrast, the level of Akt phosphorylation at Ser473 was not apparently upregulated by KCTD20 (Figure 3A).

### Intracellular localization of KCTD20 is similar to BTBD10

**Figure 4:**
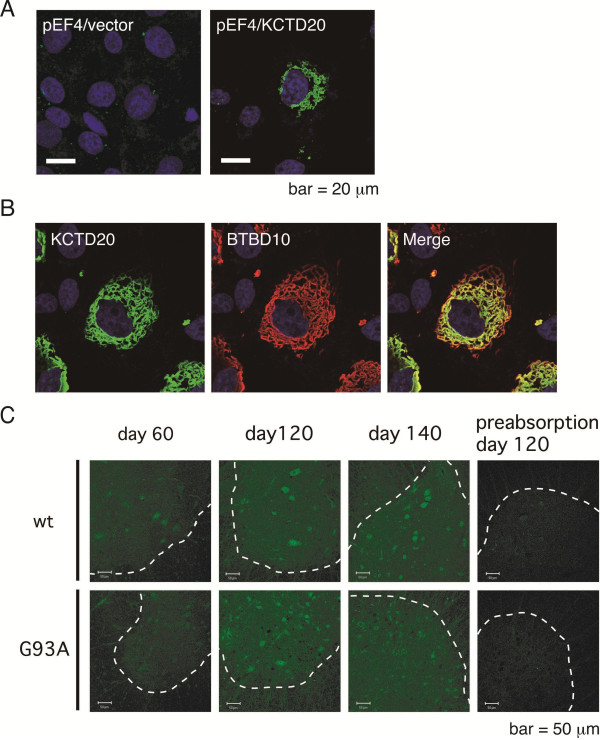
**Intracellular localization of KCTD20 or expression level of KCTD20 in mouse spinal cord anterior horn. A**, His-Xpress-KCTD20 was expressed in COS7 cells by transfection of pEF4-KCTD20 endocing HisXpress-tagged KCTD20. The backbone pEF4 vector was similarly transfected as a negative control. Transfected cells were fixed with 4% paraformaldehyde and immunostained with anti-Xpress antibody as a primary antibody and FITC-conjugated anti-mouse IgG antibody as a secondary antibody. The scale bar indicates 20 μm. **B**, COS7 cells were transfected with pEF4-KCTD20 and pCAGGS-BTBD10 and fixed at 48 hr after transfection. The cells were immunostained with Xpress or BTBD10 antibody as a primary antibody and FITC-conjugated anti-mouse IgG antibody or TexasRed-conjugated anti-rabbit IgG antibody as a secondary antibody, respectively. **C**, Frozen sections of spinal cords of G93A-SOD1-Tg mouse or wild-type littermate were immunostained with KCTD20 antibody. Rightmost pictures in each series (indicated as day 120 preabsorption) were immunostained using KCTD20 antibody, preabsorbed with the antigen peptide. Areas, surrounded by dashed lines, represent spinal ventral horns. The scale bar indicates 50 μm.

BTBD10 intracellularly localizes in cytoplasm and shows a unique filamentous structure [9]. In the present study, KCTD20 also localized in cytoplasm and had a filamentous structure (Figure 4A). To examine whether KCTD20 colocalizes with BTBD10, we coexpressed His-Xpress-tagged human KCTD20 and BTBD10 in COS7 cells and immunostained them using Xpress and BTBD10 antibodies. KCTD20 and BTBD10 colocalized in the same filamentous structure (Figure 4B).

### Expression of KCTD20 is not downregulated in motor neurons in ALS mice

Decreased expression of BTBD10 has been suggested to cause motor neuron death via the downregulation of the level of phospho-Akt [11]. Immunohistochemical analysis of frozen sections of mouse spinal cords with the KCTD20 antibody has shown that KCTD20 is expressed in motor neurons in anterior horns of spinal cords (Figure 4C). In a previous study [11], levels of BTBD10 expression were found to be downregulated in motor neurons in the spinal cords of G93A-SOD1 transgenic mice at advanced stages of ALS. We therefore examined levels of KCTD20 expression in the same G93A-SOD1 transgenic mice. At an early symptomatic stage (Day 60), the level of KCTD20 expression in G93A-SOD1 transgenic mouse motor neurons was similar to that in motor neurons in wild-type littermates. Although the level of BTBD10 expression was decreased in motor neurons of G93A-SOD1 transgenic mice, compared with that of wild-type littermates, at 120 days [11], the level of KCTD20 expression was not decreased at 120 days or 140 days (Figure 4C).

## Discussion

In the current study, we identified KCTD20, an isoform of BTBD10, as a novel putative Akt or PP2A-interacting protein. Based on the result that overexpression of KCTD20 increased the level of Akt phosphorylation at Thr308, it is highly likely that similarly to BTBD10, KCTD20 positively regulates Akt (Figure 3). On the other hand, overexpression of KCTD20 or BTBD10 did not apparently increase the level of phosphorylation of Akt at Ser473 (Figure 3). The previous study also showed that overexpression of BTBD10 only weakly increased the level of phosphporylation of Akt at Ser473 while it increased the level of phosphporylation of Akt at Thr308 in a definitive manner [9].

Phosphorylation of Akt at Thr308 and Ser473 is catalyzed by different kinases, i.e., PDK-1 and PDK-2 (or Ser473 kinase), respectively [1,2]. Similarly, phosphatases involved in the dephosphorylation of Akt at Ser473 may be different from those required for dephosphorylation of Akt at Thr308. The putative phosphatases of Akt have been proposed to be PP2A [15] and PHLPP1 (or PHLPP2) [16,17]. Zhuo et al. has recently reported that CSTP1 is a specific phosphatase of Akt at Ser473 [18]. It is possible that KCTD20 and BTBD10 may preferentially interact with the phosphatase of Akt at Thr308.

Phosphorylations of Akt at both Thr308 and Ser473 are necessary for the full activation of Akt [1,2]. However, it has also been suggested that phosphorylation at Ser473 may be unnecessary for activation of the majority of downstream Akt targets, such as TSC2, GSK3, and the TORC1 effectors, S6K and 4E-BP1 but necessary for FoxO1/3a [19,20]. Therefore, dysregulation of the function of KCTD20 and BTBD10 may affect many cellular processes by changing the phosphorylation of Akt at Thr308.

Akt may act as an inhibitor of neuronal apoptosis and loss-of-function of Akt may contribute to the pathogenesis of ALS. In support of this hypothesis, it has been shown that levels of phospho-Akt are decreased in motor neurons of spinal cords of ALS [7,8], administration of IGF-1 or VEGF, which activates Akt, prolongs the lifespan of ALS model mice [21], and VEGF-deficient mice show an ALS-like phenotype [22].

The level of BTBD10 expression has recently been shown to be downregulated in motor neurons in sporadic human ALS cases [11]. Notably, the level of BTBD10 expression is downregulated only in motor neurons that contain TDP-43 aggregates [12]. In a previous study [11], BTBD10 expression was also shown to be downregulated in motor neurons in G93A-SOD1 mice at advanced ALS stages. On the other hand, KCTD20 expression was not downregulated in motor neurons in G93A-SOD1 mice at advanced ALS stages (Figure 4). This finding suggests that KCTD20 is not involved in the ALS pathogenesis in contrast to BTBD10. However, this needs to be confirmed by examining whether KCTD20 expression is unchanged in motor neurons in other ALS mouse models (e.g., mutant TDP-43 transgenic mouse or FUS transgenic mouse) and ALS patients.

Levels of KCTD20 expression in a majority of non-nervous tissues were found to be equal to or higher than those in nervous tissues (Figure 1B), whereas levels of BTBD10 expression have previously been shown to be much lower in the majority of non-nervous tissues than nervous tissues [9]. This finding on tissue distribution suggests that KCTD20 plays a major role as an Akt activator in these non-nervous as well as nervous tissues and dysregulation of KCTD20 may be linked to diseases involving these tissues. Detailed characterization of the function of KCTD20 will serve as an important hint to the understanding of Akt-related biological events.

## Conclusions

KCTD20 is a novel positive regulator of Akt phosphorylation at Thr308. KCTD20 may be involved in cellular process via Akt in non-nervous and nervous tissues.

## Methods

### Cell culture

COS7 cells or motor neuronal cell NSC34 cells were cultured in Dulbecco’s modified Eagle’s medium (Wako Pure Chemical Industries, Osaka, Japan) supplemented with 10% fetal bovine serum (Hyclone, Logan, UT, USA).

### Antibodies

A rabbit polyclonal antibody to mouse KCTD20 was generated by immunization with a synthetic peptide, LNAPLSQMAPNDFQD, corresponding to the C-terminal 15-amino-acid peptides of mouse KCTD20, conjugated to Keyhole Limpet Hemocyanin (Sigma-Ardrich, Saint-Louice, MO, USA). Phospho-Akt (Thr308) (#4056), phospho-Akt (Ser473) (#4060), Akt (#9272), or GAPDH were purchased from Cell Signaling Technology (Danvers, MA, USA). Anti-Xpress antibody or anti-actin antibody was purchased from Invitrogen (Carlsbad, CA, USA) or SIGMA (St. Louis, MO, USA), respectively. Anti-GST monoclonal antibody was purchased from Upstate Biotech (Charlottesville, VA). HRP-conjugated anti-mouse IgG antibody or anti-rabbit IgG antibody, used as the secondary antibody, was purchased from Bio-Rad (Hercules, CA).

### Plasmids

Human KCTD20 cDNA was obtained from human testis cDNA (BioChain, Newark, CA, USA) using a primer set, a sense primer 5′-CGGGATCCATGAATGTTCACCGTGGCAG-3′ and an antisense primer 5′-CGAATTCCTAATCCTGAAAGTCGTTAGAAGC-3′. Mouse KCTD20 cDNA was amplified by RT-PCR (High-fidelity RT-PCR kit, Takara) using total RNA isolated with ISOGEN (Wako) from NSC34 cells with a sense primer 5′-CGGGATCCATGAATGTTCACCAGGGCAG-3′ and an antisense primer 5′-GGAATTCCTAATCTTGAAAGTCATTCGGAGC-3′. The cloned cDNAs were subcloned into pEF4 His-Xpress vector at a cloning site BamHI/EcoRI. The plasmids encoding the catalytic subunit of PP1A (PP1Ac), the catalytic subunit of PP2A (PP2Ac), Akt, or BTBD10 were constructed, as described previously [9].

### GST-pulldown assay

COS7 cells, seeded onto 60-mm dishes, were transfected with expression vectors by LipofectAMINE (Invitrogen) and Plus reagent (Invitrogen) following the manufacturer’s protocol. The transfected cells were harvested at 48 hr after transfection and lysed with a lysis buffer [20 mM HEPES (pH 7.5), 150 mM NaCl, 1 mM dithiothreitol, 1 mM EDTA, 0.5% Triton X-100, and protease inhibitor cocktail] by pipetting and sonication. After centrifugation, the supernatants were precleared using sepharose 4B beads (GE Healthcare Bio-Sciences Corp., Piscataway, NJ) for 7 hr followed by GST-pulldown using glutathione beads (GE Healthcare Bio-Sciences Corp.). The pulled-down beads were washed five times with protease inhibitor free lysis buffer and subjected to SDS-PAGE followed by immunoblotting with anti-Xpress antibody or anti-GST antibody.

### Immunohistochemistry

Frozen sections of spinal cords from G93A-SOD1 transgenic miceor wild type littermates were immunostained with KCTD20 antibody as a primary antibody (0.01 mg/ml) and FITC-conjugated anti-rabbit IgG antibody as a secondary antibody (1:200).

## Abbreviations

KCTD20: Potassium channel tetramerization protein domain containing 20; PP1A: Protein phosphatase 1A; PP2A: Protein phosphatase 2A; PIP3: Phosphatidylinositol-3,4,5-trisphosphate; PDK-1: 3-phosphoinositide-dependent kinase-1; ALS: Amyotrophic lateral sclerosis; SOD1: Superoxide dismutase 1; TDP-43: Transactive response DNA binding protein 43 kDa.

## Competing interests

Both authors declare that they have no competing financial interests.

## Authors’ contributions

MN designed and performed the experiments, and analyzed the data, and wrote the manuscript. MM directed the study, designed the experiments, analyzed the data, and wrote the manuscript. Both authors read and approved the final manuscript.
